# Characterization of B_0_-field fluctuations in prostate MRI

**DOI:** 10.1088/1361-6560/abbc7f

**Published:** 2020-11-02

**Authors:** Lebina Shrestha Kakkar, Muhammad Usman, Simon Arridge, Alex Kirkham, David Atkinson

**Affiliations:** 1 Centre for Medical Imaging, University College London, Foley Street, London, UK; 2 Centre for Medical Imaging Computing, University College London, High Holborn, London, UK; 3 Radiology Department, University College Hospital, Euston Road, London, UK

**Keywords:** B_0_-field, prostate MRI, diffusion MRI, mpMRI, prostate phantom, CEST, MRS

## Abstract

Multi-parametric MRI is increasingly used for prostate cancer detection. Improving information from current sequences, such as T2-weighted and diffusion-weighted (DW) imaging, and additional sequences, such as magnetic resonance spectroscopy (MRS) and chemical exchange saturation transfer (CEST), may enhance the performance of multi-parametric MRI. The majority of these techniques are sensitive to B_0_-field variations and may result in image distortions including signal pile-up and stretching (echo planar imaging (EPI) based DW-MRI) or unwanted shifts in the frequency spectrum (CEST and MRS). Our aim is to temporally and spatially characterize B_0_-field changes in the prostate. Ten male patients are imaged using dual-echo gradient echo sequences with varying repetitions on a 3 T scanner to evaluate the temporal B_0_-field changes within the prostate. A phantom is also imaged to consider no physiological motion. The spatial B_0_-field variations in the prostate are reported as B_0_-field values (Hz), their spatial gradients (Hz/mm) and the resultant distortions in EPI based DW-MRI images (*b*-value = 0 s/mm^2^ and two oppositely phase encoded directions). Over a period of minutes, temporal changes in B_0_-field values were ≤19 Hz for minimal bowel motion and ≥30 Hz for large motion. Spatially across the prostate, the B_0_-field values had an interquartile range of ≤18 Hz (minimal motion) and ≤44 Hz (large motion). The B_0_-field gradients were between −2 and 5 Hz/mm (minimal motion) and 2 and 12 Hz/mm (large motion). Overall, B_0_-field variations can affect DW, MRS and CEST imaging of the prostate.

## 1. Introduction

Prostate cancer (PCa) is the second largest cause of male cancer deaths in the UK (Caul and Broggio 2016) making PCa assessment a necessity. Following clinical suspicion for localised PCa, it is common practice to use diagnostic multi-parametric magnetic resonance imaging (mpMRI) combined with standardised reporting such as Likert score (Dickinson *et al* 2013) or PI-RADS version 2.1 (Turkbey *et al* 2019). mpMRI involves T2-weighted (T2W), dynamic contrast-enhanced (DCE) and diffusion weighted (DW) MRI. Although mpMRI may prevent 27% of men from having invasive biopsies, its specificity is only 41% compared to 96% for the biopsies (Ahmed *et al* 2017). Improving the quality of existing imaging sequences in mpMRI and adding extra information using other MRI techniques (such as magnetic resonance spectroscopy (MRS) and chemical exchange saturation transfer (CEST)) (Jia *et al* 2011, Li *et al* 2013, Roethke *et al* 2014) can potentially enhance PCa assessment.

Echo planar imaging (EPI) based DW-MRI sequences are an integral part of mpMRI due to their high tumour contrast and short acquisition time (Kirkham *et al* 2013, Metens *et al* 2012). However, they often exhibit shift, shears and geometric distortions in the phase encoding (PE) direction caused by a combination of low bandwidth in the PE direction and the presence of off-resonance effects, such as B_0_-field inhomogeneities and susceptibility differences at the tissue-air interfaces (e.g. rectum-prostate interface). Stretching distortions results from regions where there is a gradient of the B_0_-field in the direction of the PE direction and pile-up distortions occur when the in-plane gradient of the B_0_-field opposes the PE direction (Jezzard and Balaban 1995, Jezzard 2012).

EPI-based DWI, CEST and MRS are prostate imaging MR techniques that are affected by B_0_-fields. A B_0_-field map can be calculated from the phase differences of the two echoes of a dual-echo gradient echo scan. In a distorted EPI image, this field map can be used in a correction scheme to move the warped EPI image pixels into their correct positions. Such distortion correction methods based on the spatially varying B_0_-field maps are either simple to use (Jezzard and Balaban 1995, Jezzard 2012) and/or can correct for difficult distortions (pile-ups) (Usman *et al* 2018), especially in the prostate. However, potential temporal B_0_-field changes due to patient motion (Alhamud *et al* 2016) can result in incorrect pixel shifts across a DW dataset leading to an inaccurately computed apparent diffusivity coefficient (ADC) map—possibly hindering PCa assessments (Nketiah *et al* 2018). Temporal B_0_-field changes may cause incorrect frequency shifts in CEST (Sun *et al* 2007), whereas in MRS both temporal changes and spatially varying B_0_-fields may cause spectral line broadening (Scheenen *et al* 2007)—these result in overlapping signals leading to a loss of accuracy of the imaging method. Hence, a knowledge of the B_0_-fields is important in prostate MRI.

The purpose of this paper is to characterize B_0_-fields within the prostate by providing a measure of temporal changes in B_0_-field values (Hz) over a specific time (minutes), as well as a measure of the spatial B_0_-field values such as representative B_0_-field values within the prostate, their spatial gradients (Hz/mm) and their impact on distortions in EPI images. Our findings may inform the MR community when developing sequences and processing methods for prostate MRI, particularly those involved with DW-MRI, CEST and MRS.

## 2. Materials and methods

All experiments were performed on a 3 T Philips Achieva TX system (Philips Healthcare, Best, The Netherlands) equipped with a 16 anterior and 16 posterior channel cardiac receive coil array. Images were acquired for ten male patients and a prostate phantom to differentiate observations resulting from physiological motion. The study was approved by the London—Central Research Ethics Committee (REC# 16/LO/1440) and all subjects gave informed consent.

### 2.1. Prostate phantom

**Figure 1.**
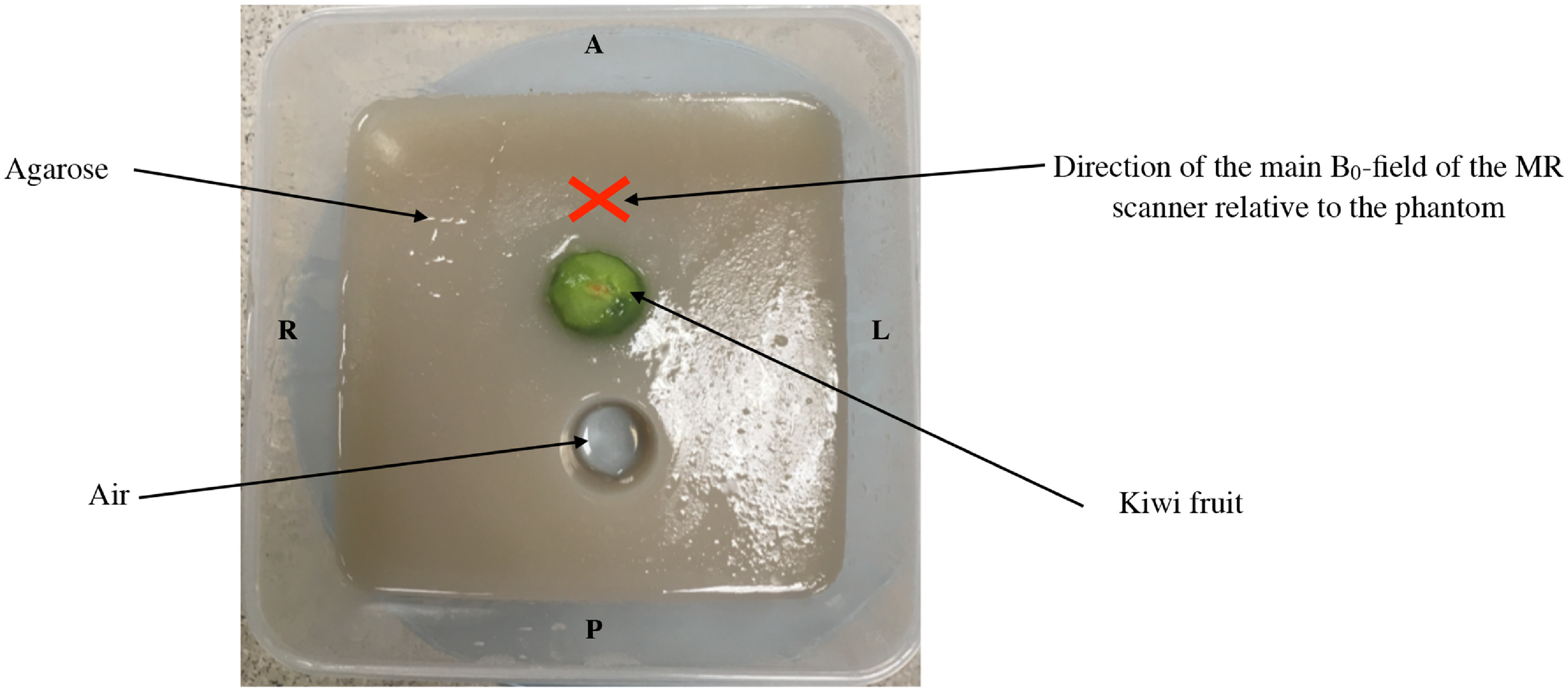
Photo of the prostate phantom. The prostate phantom consists of a peeled kiwi (the ‘prostate’) and a cylindrical air gap (the ‘rectum’) both embedded in the agarose. The phantom is scanned in a position similar to a patient lying supine in foot to head direction, i.e. kiwi is anterior to the air filled cylinder. The red cross demonstrates the direction of the main static B_0_–field of the MR scanner relative to the phantom.

50 g of agarose was stirred in 2.1 l of tap water at room temperature and was heated until the agarose dissolved. Half of the mixture was poured into a plastic container (Sainsbury’s Home Klip Lock Storage Square 5 l, dimensions 24 × 24 × 12.5 cm) and allowed to cool, whilst the remaining half was gently heated. The container contained a drinking glass (dimensions 3 × 5 cm), which was filled with weights to prevent it from floating. Similar to Bude and Adler (1995) once the first layer of agarose had lightly set, a peeled kiwi fruit (the ‘prostate’ phantom (Mueller-Lisse *et al* 2017)) was placed on top of the layer near the glass. The remaining mixture was poured into the container and allowed to set overnight; 4–5 h prior to the experiment, the glass was removed to create the air filled ‘rectum’. The prostate phantom is shown in figure 1.

### 2.2. Subjects

Ten male patients (median (range) weight 84 (68–98) kg and age 68 (57–79) years old) were recruited from the clinical prostate imaging pathway. Patients were placed in a supine, feet first position into the scanner and imaging was carried out during free breathing for all patients. No antispasmodic agent was administered. Patient 2 had been previously treated with High Intensity Focused Ultrasound (HIFU) therapy and patient 3 had eaten 15 min prior to the scanning session.

### 2.3. Imaging

Temporal and spatial characterization of B_0_-fields were carried out using dynamic fast dual-echo gradient echo (FFE) sequences. Axial images were acquired using sequences with the following parameters: flip angle = 6^°^ , first echo time (TE) = 4.6 ms, TE difference = 2.3 ms, relaxation time (TR) = 8.6 ms, axial field-of-view (FOV) = 230 × 230 mm^2^, where the number of slices acquired depended on the prostate size of the patient, slice thickness = 4 mm, volume shim and right to left PE direction. The dynamic B_0_-field maps were automatically computed by the scanner in Hz.

Temporal B_0_-field variations were evaluated for different time scales. A single slice 2D scan acquired every 1.75 s over 53 s was used as a short time scale. For longer time scales (≥150 s), multiple 3D sequences with varying SNR were compared and only one was chosen for subsequent analysis. SNRs were varied by changing the bandwidth and voxel size and the resultant SNR change was estimated by the Philips scanner. Table 1 summarises the sequence parameters for the different gradient echo sequences.

**Table 1.** Summary of the different dynamic dual-echo gradient echo sequences in the order of increasing SNR. The SNR factors relative to the first sequence are predicted by the scanner when changing the sequence parameters.

Sequence no.	Scan type	Acquired image resolution (mm^3^)	No. of slices	Bandwidth/mm (Hz/mm)	No. of dynamics	Time for each dynamic (s)	SNR factor relative to Sequence 1
1	2D	2× 2× 4	1	433	30	1.75	1.0
2	3D	1× 1× 4	15–23	321	6	33.2	2.7
3	3D	2× 2× 4	15–23	433	9	18.5	4.4
4	3D	2× 2× 4	15–23	160	9	19.5	7.6

B_0_-field maps were also related to the distortions in EPI based DW-MRI images. As the distortions are linked to the imaging gradients and not the diffusion encoding gradients, two EPI sequences with only the *b* = 0 s/mm^2^ of a DW sequence were used with opposite PE gradients: One with anterior to posterior PE direction (PE:AP) and vice versa (PE:PA). The remainder of the DW sequence parameters are: resolution = 2 × 2 × 4 mm^3^, FOV = 180–220 × 180–220 × 4 mm^3^, SENSE factor = 2, TR = 2000 ms, TE = 80 ms, bandwidth in the PE direction ∼20.8–22.4 Hz/pixel (or 10.4–11.2 Hz/mm). For reference purposes, an axial T2W image was acquired using a turbo spin echo sequence with the following parameters: resolution = 2 × 2 × 4 mm^3^, FOV = 180–220 × 180–220 × 60–92 mm^3^, SENSE factor = 2, TR = 4700 ms, TE = 100 ms.

### 2.4. Image analysis

A single slice of the 3D gradient echo magnitude image from sequence 3 was chosen such that it was closest to the single 2D slice from sequence 1. ROIs were placed using the magnitude images and the reference T2W image to best visualise the prostate position by a radiologist with 25 years of experience. Inspection of all datasets did not suggest prostate motion caused the ROI to include non-prostate areas. However if severe physiological motion was to occur, the ROIs could be shifted out of the prostate introducing errors into the analysis. The B_0_-field values within the ROI were extracted for each case to characterise the temporal B_0_-field variation.

The spatial B_0_-field variation across the prostate was characterized in three separate slices: the original mid-axial slice and two additional slices inferior and superior to the mid-axial slice. The centre-to-centre separation between each of the slices is 8 mm. The pixelwise B_0_-field values within the ROI of the slices were extracted from the first dynamic of Sequence 3 in table 1.

Line profiles within the prostate ROI (posterior to anterior (PA) and right to left (RL)) were also drawn to evaluate the B_0_-field values across the prostate for the mid-axial slice. Additionally, gradients of the B_0_-field in the anterior-posterior direction were computed for the prostate ROIs at the three slices. Gradient values at the posterior edge of the prostate (where B_0_-field varied considerably) were recorded by selecting the last three rows of pixels within the ROI at the posterior of the prostate. A two-sided Wilcoxon signed rank test was used to determine whether the B_0_-field gradients at the posterior edge was significantly different from zero and the sign of the gradient was noted. Distortions in the reverse phase encoded DW images were then compared to these B_0_-field gradients using the T2W image as reference.

## 3. Results

**Figure 2.**
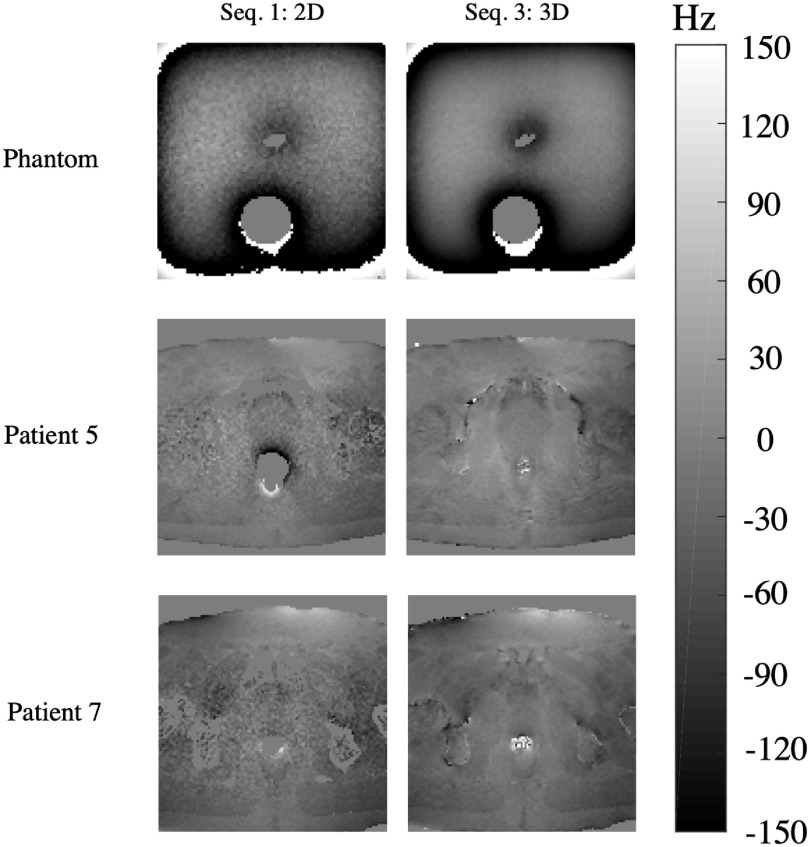
Images of B_0_-field map for a single axial slice of the phantom (first row) and two example patients (patients 5 (second row) and 7 (third row)). Images displayed are from the sequences 1 and 3 from table 1.

Figure 2 displays example B_0_-field maps for Sequence 1 and Sequence 3 from table 1. The B_0_-field map shows a large variation in the B_0_-field across the image plane and is dependent on the material/tissue type. It also visually demonstrates that low SNR leads to an apparent increase in B_0_-field variation.

**Figure 3.**
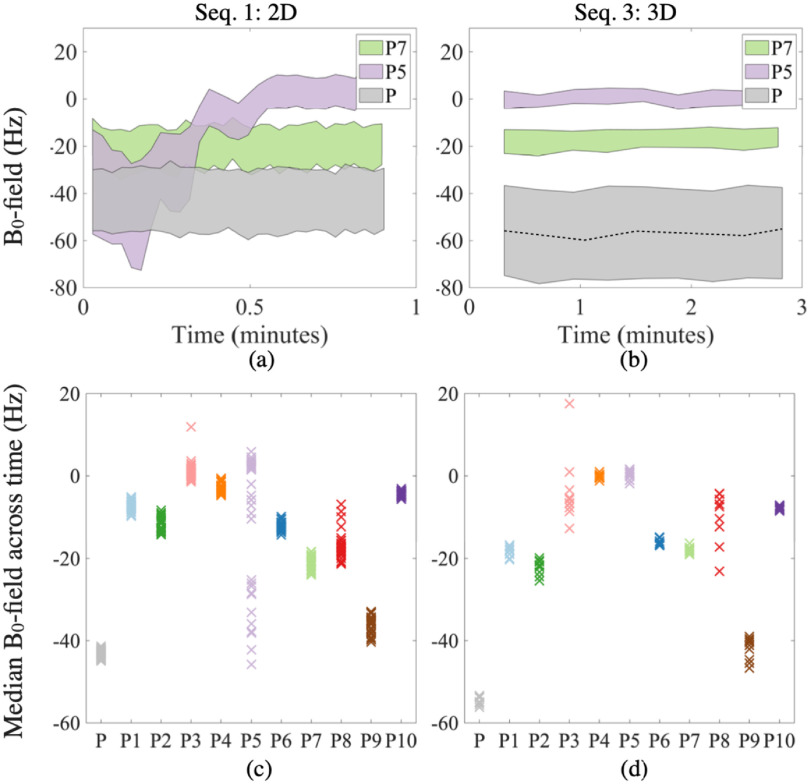
Temporal changes in B_0_-field values for the phantom (P) and patients 5 and 7 (P5 and P7) using Sequence 1 (2D, 0.9 min, left column) and sequence 3 (3D, 2.8 min, right column). Top row shows the 25th and 75th percentile of B_0_-field values within an ROI as a function of time, where time 0 is the acquisition of the first image. The median value is also plotted as a dashed line for the phantom in Sequence 3. The bottom row plots the median B_0_-field values at every time point for the phantom and each subject.

The first row in figure 3 demonstrates the changes in B_0_-field within the ROI of the phantom and two example patients (patients 5 and 7) over the duration of the dynamic sequences 1 and 3 from table 1. Sequence 1 of figure 3 shows that B_0_-fields are consistent throughout the duration of the sequence (51 s) for the phantom and patient 7, however for patient 5, large fluctuations initially occur potentially due to rectal size changes. As expected, the SNR increases for the 3D sequence and the range of measured B_0_-field values reduces within the prostate. While only results from two example patients are shown here, the measured distribution was consistently smaller in the 3D sequence for all patients. Unlike the patients, the measured B_0_-field range is higher in the 3D sequence for the phantom, possibly because the underlying signal from the kiwi phantom was lower in the 3D sequence.

The second row in figure 3 summarises the changes in the median B_0_-field of the ROI across time for the phantom and all patients. The largest range of the median B_0_-field of the ROI is observed for patient 5 (52 Hz across ≈1 min) in Sequence 1. However, the range of the median B_0_-field values, which indicates the temporal changes, are much lower across other patients—their minimum—maximum ranges are: 2.5–14 Hz (Sequence 1, i.e. over a duration of 0.9 min) and 1.4–19 Hz (Sequence 3, i.e. across 2.8 min). The B_0_-field ranges are smaller for the phantom (between 2.0 and 3.6 Hz over a duration of $\lt$3 min) regardless of the sequences used.

**Figure 4.**
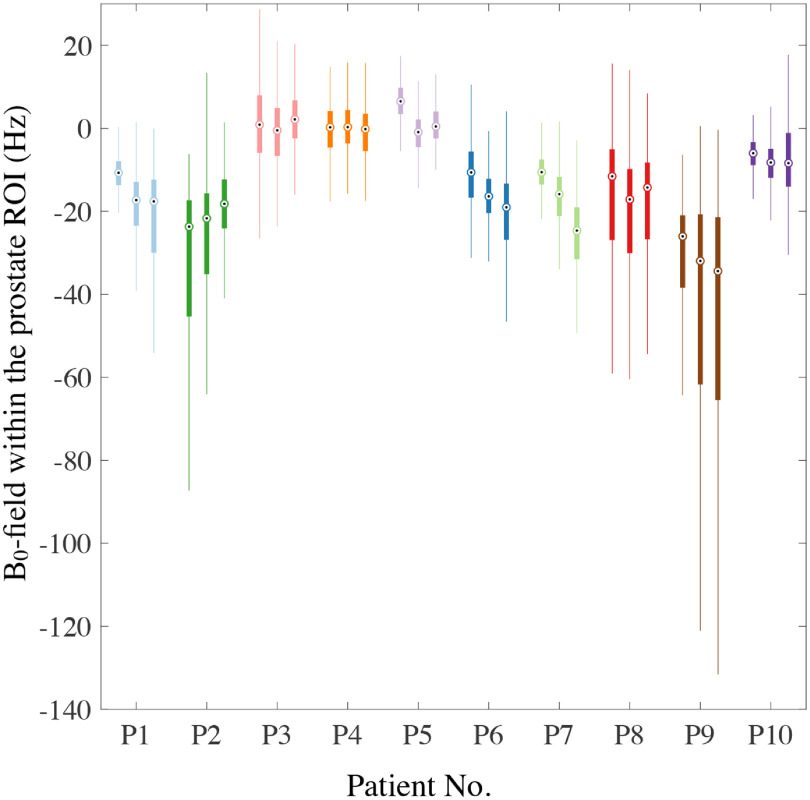
B_0_-field values within the prostate volume from the first dynamic scan of Sequence 3 in table 1 for all patients. The B_0_-field values at the ROIs of the three chosen slices for each patient are displayed as a set of three box and whiskers of the same colour, where the boxes represent the 25th, 50th and 75th percentile of the B_0_-field values at the ROIs and the whiskers extend to cover 99.3% of the B_0_-field values at the prostate. The three slices are named inferior, mid-axial and superior prostate with a centre-to-centre separation of 8 mm.

Figure 4 summarises the B_0_-field distribution within the prostate ROI for all patients for the first dynamic scan of Sequence 3. The minimum and maximum median B_0_-field values across the prostate are between −25 and 6.3 Hz, respectively, for all patients except patient 9. The interquartile ranges (IQRs) are ≤18 Hz for all patients except patient 2 (contains fluid filled region following HIFU treatment) and patients 8 and 9 (large bowel motion were observed during Sequence 3 and could also have occurred within the first dynamic of Sequence 3 for both patients), where the interquartile ranges are as high as 44 Hz.

**Figure 5.**
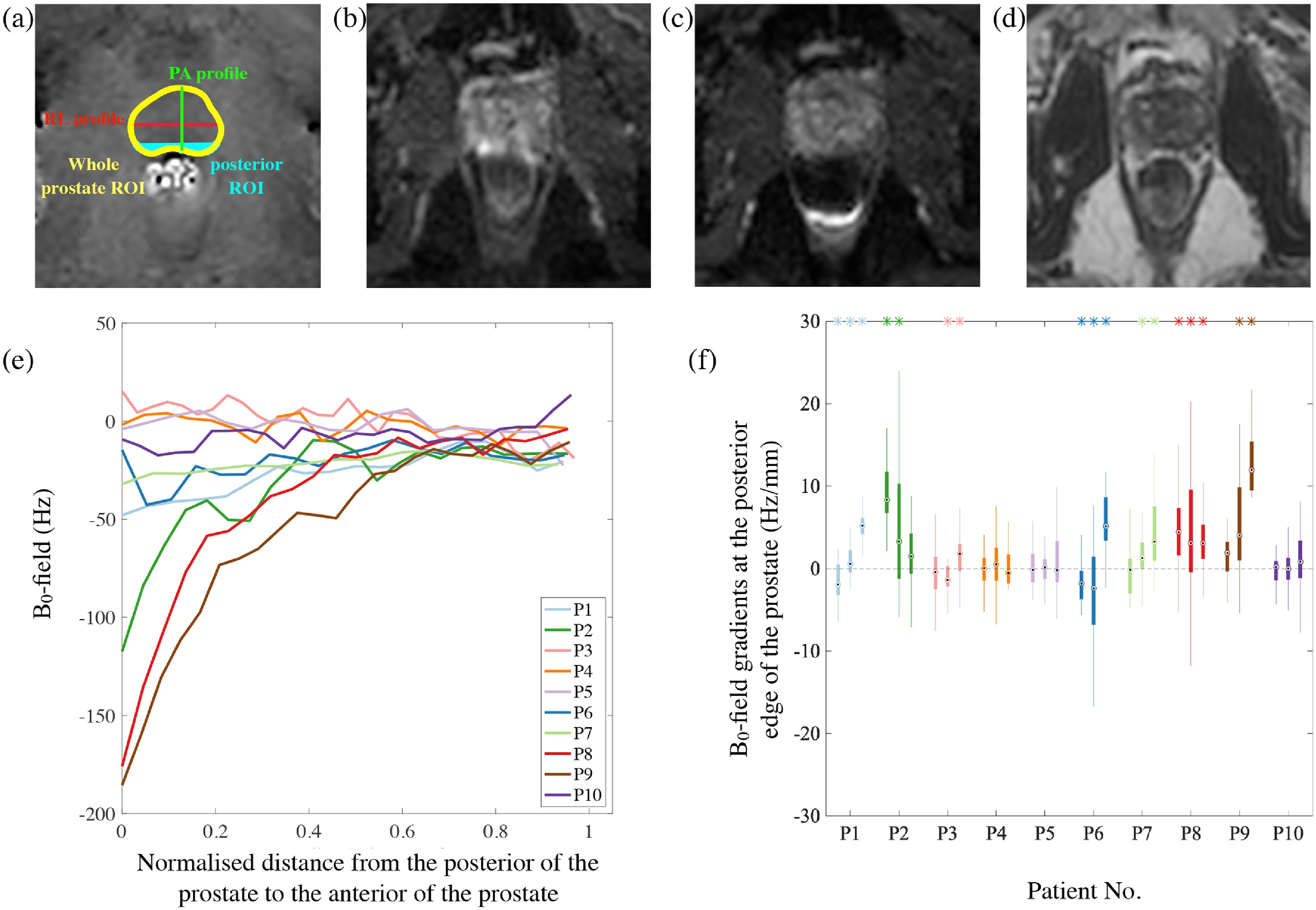
B_0_-field gradients and their effect on DW-MR images of the prostate. (a) An example axial B_0_-field map calculated from a 3D gradient echo (Sequence 3 in table 1) (b) and (c) examples of the distorted non-DW image acquired with the PE:AP and PE:PA directions, respectively and (d) the reference T2-weighted image of the prostate, for patient 7. A prostate is outlined (in yellow) in (a), along with the PA and RL profiles (green and red lines, respectively), as well as the posterior edge region of the prostate (shaded in cyan). The B_0_-field profiles for the mid-axial slice are plotted for all patients and are displayed in (e). (f) The compact box and whisker plot displays the range of B_0_-field gradients (similar to figure 4) that are at the posterior edge of the prostate for the three prostate slices and a coloured * at the top of the plots indicate whether the median gradient values are significantly different from zero ($p\lt 0.05$).

Figure 5(a) displays an example B_0_-field map, example line profiles and ROIs that are drawn on the prostate. Figure 5(b) and c demonstrate an example of the effect of B_0_-field gradients and their effects on two reverse phased encoded non-diffusion weighted images in comparison to the reference T2W image (figure 5(d)). Figure 5(e) shows the B_0_-fields along the PA profile for the mid-axial slice of the prostate, where the B_0_-fields increase/decrease until they reach similar B_0_-field values at the anterior of the prostate. In contrast, the B_0_-field profile along RL were generally flat with small fluctuations (results not shown).

Figure 5(f) displays the range of numerical gradients at the posterior edge of the prostate for the same slices from figure 4. The values range approximately from −20 to 20 Hz/mm. Significantly positive, negative and zero B_0_-field gradients are observed for ∼50%, $\lt$15% and ∼30% of the dataset, respectively. Additionally, for some patients (patients 1, 3 and 6), the polarity of the gradients are varied for different slices of the same prostate. Visual comparison of the B_0_-field gradients to the distortions observed in the DW images with respect to T2W images show negative B_0_-field gradients correspond to pile-up distortions and positive gradients correspond to stretching distortions when imaging in PE:AP direction and vice versa in the PE:PA direction. As expected, patients 4, 5 and 10 have small B0-field gradients and show little distortion in their DW images.

## 4. Discussion

In this study, we characterized the temporal and spatial variations in the B_0_-field. The temporal B_0_-field changes in the prostate are higher in patients than in the phantom. Typically, B_0_-field values fluctuated by 1–19 Hz over a time period of $\lt$3 min and in-plane median B_0_-field values at the prostates were between −25 and 6 Hz (with an interquartile range of up to 18 Hz) for cases of very little to no bowel motion. In EPI based DW-MRI dataset acquired with a PE bandwidth of 21 Hz/pixel (10.5 Hz/mm) on a 3 T MR scanner, these correspond to shifts between 0.1-0.9 pixels or 0.1–1.8 mm (compared to $\lt$0.2 pixels ($\lt$0.3 mm) for the phantom) between subsequent DW measurements and an additional shift of $\lt$1 pixel ($\lt$2 mm) in each DW measurement. For larger B_0_-field changes (for instance when fluid filled lesion was included in the prostate ROI or when large bowel motion occurred), shifts between subsequent DW measurements can be 1–2.5 pixels or 2–5 mm (with an additional average shift of ≈2 pixels (≈4 mm) within the prostate per measurement), resulting in misaligned ‘corrected’ DW data leading to miscalculation of ADC maps.

In the 30 slices analysed over the 10 patients as part of this study, stretching occurred more frequently than pile-up distortions at the posterior edge of the prostate when the patients were imaged supine, feet first and with phase encoding in the AP direction than when the phase encoding direction was reversed. Stretching can be easier to correct (Jezzard and Balaban 1995, Embleton *et al* 2010), or less harmful to image interpretation and so consideration of the phase encode direction may be beneficial.

Other than EPI based DW-MRI, the B_0_-field changes at the prostate area can potentially affect other prostate MR modalities. For instance, in CEST, heterogeneous spatial B_0_-fields can alter the z-spectrum but can be corrected using computed B_0_-field maps from pre-acquisition methods (Kim *et al* 2009, Schuenke *et al* 2017). However, CEST imaging is lengthy (≈3–6 min (Evans *et al* 2019, Liu *et al* 2019) for a single slice). Temporal B_0_-field changes of 30–50 Hz (0.23–0.40 ppm) spanning 1–3 min (observed in figure 3 on a 3 T MR scanner) and potential system drift (≈10 Hz) (Liu *et al* 2019, Windschuh *et al* 2019) may lead to wrongly corrected z-spectra, possibly increasing the chances of overlapping CEST signals from amides (≈3.5 ppm) and fast exchanging amines (≈3 ppm) and reducing the specificity of the method (Zhang *et al* 2018) to detect protein levels that are linked to PCa (Jia *et al* 2011).

Another important prostate MR modality is MRS. If the B_0_-field within the prostate are shimmed perfectly to allow accurate water and fat suppression, then the spectral data should show four frequency peaks: choline-containing compounds (3.2 ppm), polyamines (3.1 ppm), creatine (3.0 ppm) and citrate (2.5–2.8 ppm) (Li *et al* 2013). However, our findings suggest that after volume-based shimming, B_0_-field values can change up to 0.15 ppm (19 Hz) within 1–3 min and the range of B_0_-field values within the prostate could be up to 0.14 ppm (18 Hz) for minimal bowel motion. For large bowel motion, the values are much higher (≥0.23 ppm or ≥30 Hz over a duration of ≈1–3 min from figure 3)and ≤0.35 ppm (≤44 Hz) from figure 4) spatially. These may cause spectral line broadening of the metabolites preventing accurate assessment of the citrate and choline concentrations—the main metabolites for determining PCa.

In this study, we purposely used realistic imaging parameters. Even with the largest pixel size of 2 mm in the B_0_ map, acquisition times were too long to correlate with the breathing and cardiac cycles. However, temporal B_0_-field fluctuations were lower for the stationary phantom suggesting that physiological motion affects the prostate.

No antispasmodic agent was administered for this study. It is possible that antispasmodics, as often used for clinical scans to reduce bowel motion, could reduce the B_0_-field variations. However, the effectiveness of the drug can be variable and short-lived (Roethke *et al* 2013, Slough *et al* 2018), hence we would still expect some variations in B_0_-fields near the rectum area post administration of antispasmodic agents.

A phase array coil was used for prostate imaging in this study. Prostate imaging is also possible through the use of endorectal coils (ERC) with PFC or barium sulfate to reduce susceptibility differences (Rosen *et al* 2007). They may offer lower spatial field variation and lower temporal field variation (Husband *et al* 1998) but at the expense of patient discomfort. A recent comparison study suggested that there is not much difference in cancer detection using either a body phase array coil or the ERC (Tirumani *et al* 2019).

Recent heavy activity on the MR scanner could potentially make our results specific to the Philips Achieva MR scanner. A frequency drift of ∼10 Hz, caused by heating effects, can be expected on a 3 T Philips MR scanner when using rapid gradient switching sequences such as EPI in combination with diffusion gradients associated with high b-values (Liu *et al* 2019, Vos *et al* 2017). If the frequency is not re-adjusted, the effect is a constant offset to the B_0_-field. This does not cause image distortions but in EPI leads to an image shift in the phase-encode direction. However, our B_0_-field maps (acquired with FFE sequences—a less intense sequence than EPI) show temporal variations of $\gt$10 Hz suggesting that our findings would not change regardless of the drifts. Additionally, it would be interesting to perform this study on other MR scanners to test the reproducibility of our results.

Our study produced a prostate phantom to simulate the artefacts in DW-MRI based on B_0_ variations in the absence of physiological motion. The phantom geometry resembled an axial slice of the prostate and created similar B_0_-field maps and resultant distortions. Although the measured T1 and T2 of the agar (T1 ∼1800 ms and T2 ∼60 ms) and kiwi regions (T1 ∼1600-1900 ms and T2 ∼200–400 ms) in the phantom (data not shown) was not very similar to the prostate (T1 ∼1400–1700 ms and T2 ∼80 ms) and its surrounding organs (T1 ∼900–1500 ms and T2 ∼27–44 ms) (Bojorquez *et al* 2017), we do not expect these values to affect the B_0_-field maps and distortions. This phantom is easy to create, similar to (Bergen *et al* 2020), and may be useful for testing implementations of new DW-MR sequences (Hutter *et al* 2017, Kakkar *et al* 2017) on clinical scanners.

Finally, we would like to offer some guidelines that may help with prostate MRI:

• Temporal change in B_0_-field can be 1–19 Hz with minimal bowel motion and 30–50 Hz with large bowel motion over a duration of 1 and 3 min.
• Median B_0_-field values at the prostate can be between −25 and 6 Hz with an interquartile range of ≤18 Hz for minimal bowel motion and an interquartile range of ≤44 Hz for large bowel motion.
• An average B_0_-field gradient at the posterior edge of the prostate can range from −2 to +5 Hz/mm in the presence of no/small bowel motion and from +2 to +12 Hz/mm for large bowel motion.
• In this study, EPI using a phase encoding gradient that is positive in the anterior to posterior direction gave more images with stretch distortions than pile-up. As stretch distortions are easier to correct, and may be less intrusive than pile-up, further consideration of the phase encode gradient sign may be beneficial.

## 5. Conclusion

Overall, this study should inform decisions for prostate MRI applications based on CEST, MRS and, more specifically, EPI based DW-MRI—techniques that can potentially offer additional information and/or improve the quality of the mpMRI dataset for assessing the extent of PCa.
